# Oxygen reactivity with pyridoxal 5′-phosphate enzymes: biochemical implications and functional relevance

**DOI:** 10.1007/s00726-020-02885-6

**Published:** 2020-08-25

**Authors:** Giovanni Bisello, Carmen Longo, Giada Rossignoli, Robert S. Phillips, Mariarita Bertoldi

**Affiliations:** 1 Department of Neuroscience, Biomedicine and Movement Sciences, Section of Biochemistry, University of Verona, Verona, Italy; 2 Department of Chemistry, University of Georgia, Athens, GA 30602 USA; 3 Department of Biochemistry and Molecular Biology, University of Georgia, Athens, GA 30602 USA

**Keywords:** Pyridoxal 5′-phosphate-dependent enzymes, Decarboxylase, Oxidase activity, Oxidative stress, Aromatic aldehyde

## Abstract

The versatility of reactions catalyzed by pyridoxal 5′-phosphate (PLP) enzymes is largely due to the chemistry of their extraordinary catalyst. PLP is necessary for many reactions involving amino acids. Reaction specificity is controlled by the orientation of the external aldimine intermediate that is formed upon addition of the amino acidic substrate to the coenzyme. The breakage of a specific bond of the external aldimine gives rise to a carbanionic intermediate. From this point, the different reaction pathways diverge leading to multiple activities: transamination, decarboxylation, racemization, elimination, and synthesis. A significant novelty appeared approximately 30 years ago when it was reported that some PLP-dependent decarboxylases are able to consume molecular oxygen transforming an amino acid into a carbonyl compound. These side paracatalytic reactions could be particularly relevant for human health, also considering that some of these enzymes are responsible for the synthesis of important neurotransmitters such as γ-aminobutyric acid, dopamine, and serotonin, whose dysregulation under oxidative conditions could have important implications in neurodegenerative states. However, the reactivity of PLP enzymes with dioxygen is not confined to mammals/animals. In fact, some plant PLP decarboxylases have been reported to catalyze oxidative reactions producing carbonyl compounds. Moreover, other recent reports revealed the existence of new oxidase activities catalyzed by new PLP enzymes, MppP, RohP, Ind4, CcbF, PvdN, Cap15, and CuaB. These PLP enzymes belong to the bacterial and fungal kingdoms and are present in organisms synthesizing bioactive compounds. These new PLP activities are not paracatalytic and could only scratch the surface on a wider and unexpected catalytic capability of PLP enzymes.

## Pyridoxal 5′-phosphate (PLP)-dependent enzymes are able to react with oxygen

Approximately 30 years ago, it was reported (Abell and Schloss 1991) that some enzymes that do not bind metals/cofactors typical of redox reactions are able to consume oxygen. These enzymes belong to diverse families and catalyze different chemical reactions.

The paper of Abell and Schloss represented a milestone, since it was the first time, after the identification of the oxygenase reaction catalyzed by ribulose 1,5-bisphosphate carboxylase/oxygenase (RuBisCO) (Bowes et al. 1971), that other enzymes were reported to catalyze an oxygenase side reaction and raised the issue of how such activity could occur.

Actually, the question of the reactivity with oxygen for enzymes lacking a proper metal or redox cofactor is still puzzling. It is known that molecular oxygen could be present as an excited singlet and a ground triplet state, the former being highly reactive, the latter more stable, since its reaction with organic substrates is a spin-forbidden process. This latter biradical species must be activated by a series of one-electron stepwise transfer reactions leading sequentially to superoxide anion, hydrogen peroxide, hydroxyl radical, and, finally, water (Klinman 2007). A metal or a redox cofactor like flavin can readily accomplish these subsequent steps. Flavoproteins are often considered a prototype, since the flavin cofactor is able to catalyze single-electron transfers in the absence of a metal. The spin-forbidden paradox is overcome in this class of enzymes by a single-electron transfer of a reduced flavin to the triplet state of oxygen to generate superoxide and flavin semiquinone cage radical pair, leading to different reaction pathways spanning from the dissociation to produce oxygen radicals, to a second electron transfer to form hydrogen peroxide and oxidized flavin, to the formation of a covalent hydroperoxy-flavin leading to further dissociation to hydrogen peroxide or insertion of an oxygen atom into the substrate (Mattevi 2006; Chaiyen et al. 2012; Daithankar et al. 2012).

Schloss and others studied many enzymes that have unusual and non-canonical reactivity with O_2_ (Abell and Schloss 1991; Hixon et al. 1996) and, based on the knowledge collected on RuBisCo that forms a oxygen-reactive enolate intermediate (Bowes et al. 1971), they suggested that any enzyme capable of forming carbanionic intermediates could have the propensity to react with molecular oxygen (Abell and Schloss 1991). The difference between the carbanion-forming enzymes consuming molecular oxygen and those exhibiting no susceptibility to it was proposed to lie in the accessibility of the carbanion intermediate and in the stabilization of a subsequent intermediate, a peroxide anion, occurring through protonation and/or metal coordination (Abell and Schloss 1991).

The oxygenase off-pathway activities of enzymes generating carbanionic intermediates have been designated as the so-called paracatalytic reactions with O_2_ (Christen et al. 1976; Cogoli-Greuter et al. 1979; Christen and Gasser 1980). They have been proposed to play a role in the generation of reactive species important in signaling and also in contributing to the progression of neurodegenerative diseases (Bunik et al. 2007).

One of the enzymes able to catalyze an oxygenase side reaction (identified by Abell and Schloss) was the PLP-dependent *E. coli* glutamate decarboxylase (GAD). The authors reported that GAD was able to consume O_2_ in the presence of l-glutamic acid, generating succinic semialdehyde at a rate of about 0.1% that of the main decarboxylation reaction generating γ-aminobutyric acid (GABA) (Abell and Schloss 1991). The peculiarity of this reaction is that dioxygen is not at all a substrate for PLP enzymes, whose usual substrates are amino acids, amines, and some few sugars.

**Fig. 1:**
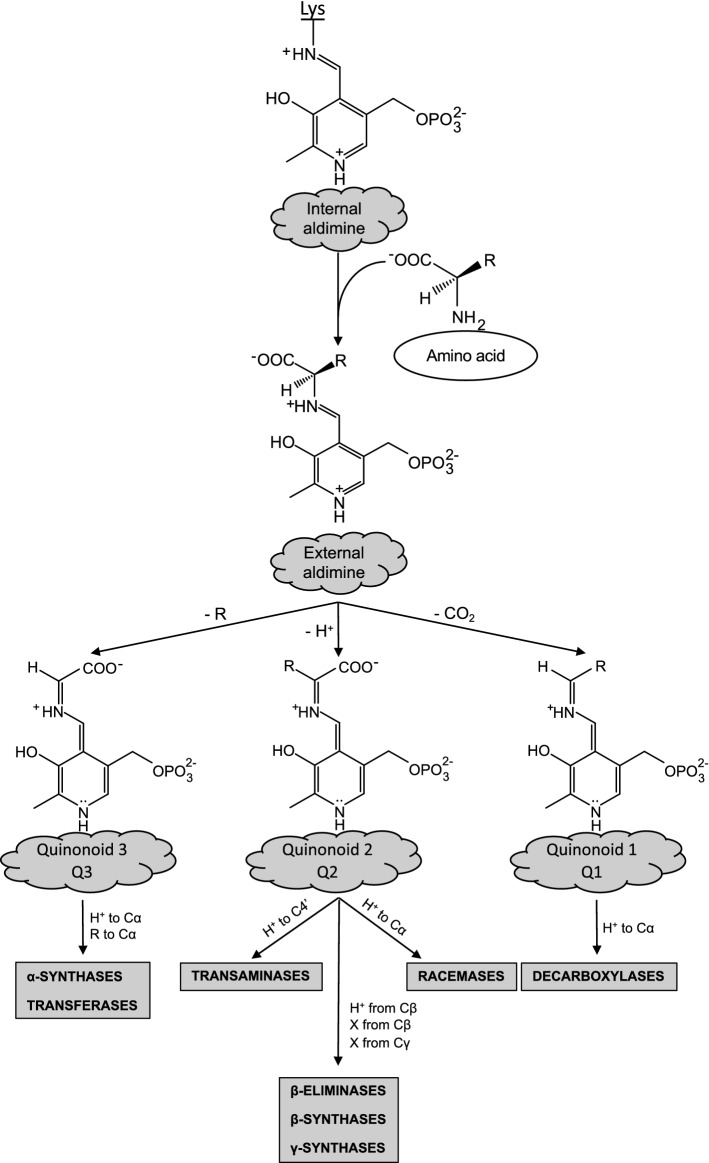
Reaction pathways of PLP enzymes. The internal aldimine is converted into the external aldimine which, subsequently, undergoes a bond breakage. The fate of the generated carbanionic intermediate, the quinonoid Q1 or Q2 or Q3, depends on reprotonation to α-carbon or to C4′ leading to the different products and PLP-dependent enzyme activities

It is known that the versatility of reactions catalyzed by PLP-dependent enzymes is largely due to the chemistry of their extraordinary catalyst. PLP is necessary for most reactions of amino acids, and it is invariably bound to the protein moiety of all PLP enzymes through a Schiff base linkage with a lysine residue forming an internal aldimine structure. In the presence of an incoming amino acid, the ε-amino group of the internal aldimine is exchanged with the α-amino group of the substrate generating the external aldimine intermediate. From this point, the different reaction pathways diverge leading to multiple non-electron transfer reactions: transamination, decarboxylation, racemization, elimination, and substitution (Fig. 1). This is accomplished by the breakage of a specific bond of the external aldimine. In 1966, Dunathan proposed (Dunathan 1966) that the scissile bond is oriented perpendicular to the plane of the imine–pyridine ring, since, in this conformation, the highest orbital overlap is achieved and, thus, the best transition state stabilization could be reached. Indeed, it is the protein scaffold that directs the positioning of the bond to be broken. This explains how reaction specificity of PLP-dependent enzymes is controlled. It follows that once the external aldimine is formed, the possibilities are that a carboxylate or a proton or a side chain will be removed. Thus, three possible different carbanionic intermediates, the quinonoid species Q1 or Q2 or Q3, are formed and, depending on the reaction that each of these quinonoids undergoes, several different pathways will be followed giving rise to the various PLP-dependent enzymatic activities (Fig. 1). This also explains the versatility of the PLP enzymes, which comprise up to 1.5% of enzymes in microbial genomes (Percudani and Peracchi 2003) and about 4% of all enzyme activities (Hoegl et al. 2018). However, the protein moiety is not able to completely abolish the intrinsic high reactivity of the cofactor, and this is the reason why PLP enzymes are promiscuous and able to catalyze, in addition to the main reaction, also side reactions with amino acids, albeit less efficiently (John 1995). Following this view, as an example, the prototype of all PLP enzymes, aspartate aminotransferase, can also perform a β-elimination and/or a racemization reaction (John and Fasella 1969; Kochhar and Christen 1988, 1992) in addition to the transaminase main reaction. This ability to catalyze one or more side reactions holds true for the majority of PLP enzymes; however, the substrates of these side reactions are always amino acids or amines. Nevertheless, given the common feature of generating carbanionic intermediates, PLP-dependent enzymes represent likely candidates also for oxygenase reactions (Fig. 1).

Since the identification of the reaction of GAD (Abell and Schloss 1991), reactivity towards O_2_ has been determined for many other PLP enzymes, as reported in the recent reviews (Hoffarth et al. 2020; Bunik et al. 2011), highlighting that this propensity is more widespread than heretofore imagined and not only confined to paracatalytic reactions.

A recent detailed overview of all reactions catalyzed by PLP enzymes involving O_2_ (Hoffarth et al. 2020) stimulates to provide more insight into the intricacy of these reaction mechanisms and to the physiological significance of this reactivity.

**Fig. 2:**
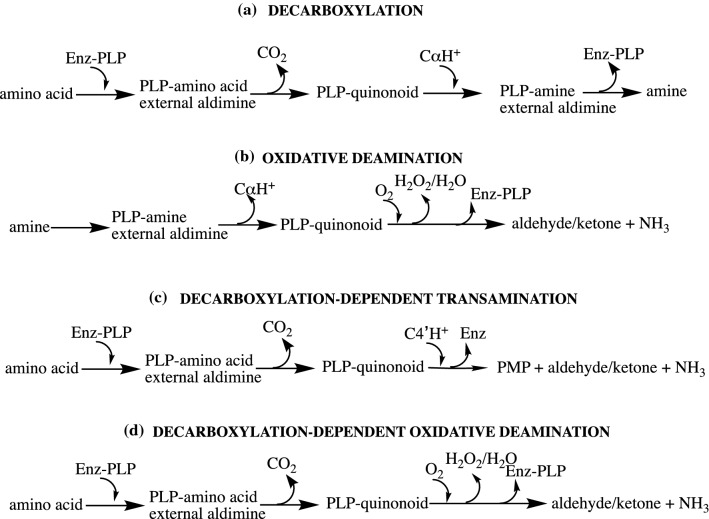
Terminology of PLP reactions. **a** Decarboxylation of an amino acid to an amine catalyzed by a PLP decarboxylase; **b** oxidative deamination of an amine into the corresponding carbonyl compound and ammonia together with O_2_ consumption; **c** decarboxylation-dependent transamination of an amino acid converted into the corresponding carbonyl compound and ammonia. Enz-PLP refers to the catalytic competent decarboxylase, Enz refers to a decarboxylase whose catalytically active coenzyme PLP has been transformed into PMP which is not a coenzyme for decarboxylation. Thus, once the PLP of the enzyme has been all transformed into PMP, the enzyme is inactivated. For this reason, the products are equimolar to PLP; **d** decarboxylation-dependent oxidative deamination of an amino acid first decarboxylated and then converted into the corresponding carbonyl compound and ammonia concomitantly with O_2_ consumption

Looking into the literature, it is evident that reactions of PLP enzymes in aerobiosis and in anaerobiosis have been annotated in different ways: decarboxylation, oxidative deamination, decarboxylation-dependent transamination, decarboxylation-dependent oxidative deamination, oxidative decarboxylation, and decarboxylating oxygenation. While the first four reactions can be typical of PLP enzymes, oxidative decarboxylation is best used for ketoacid dehydrogenases, since the products of such reactions are not aldehydes/ketones nor ammonia. For the decarboxylating oxygenation, it is used as decarboxylation-dependent oxidative deamination; however, some attention should be payed (see below). To dispel some doubts, some terminology and activity to which it refers is given to contribute and improve clarity (Fig. 2).

## The paradigmatic case of dopa decarboxylase (DDC)

**Fig. 3:**
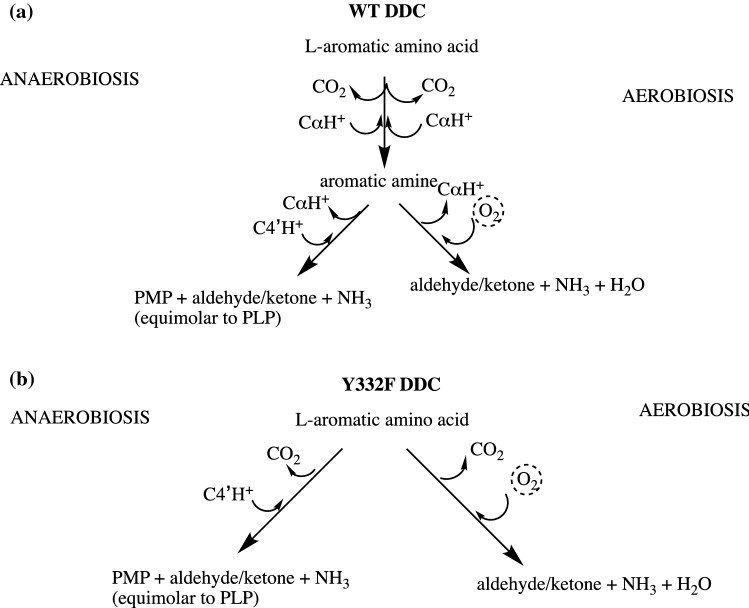
Main and paracatalytic reactions of Dopa decarboxylase. **a** The reactions catalyzed by wild-type (WT) DDC under aerobic and anaerobic conditions; **b** the reactions catalyzed by Y332F DDC under aerobic and anaerobic conditions

**Fig. 4:**
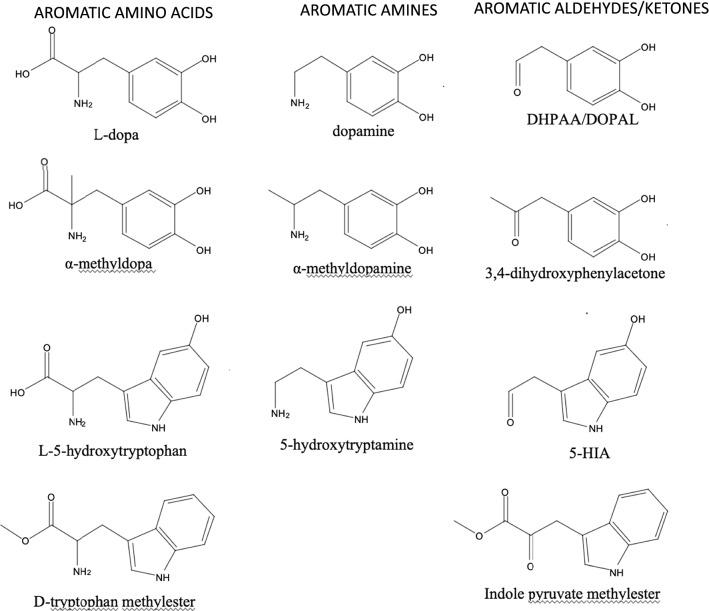
Structures of substrates and products of DDC. Aromatic amino acid substrates of the decarboxylation reaction of DDC are on the left, aromatic amines products of decarboxylation and substrates of oxidative deamination are in the center, and aromatic aldehydes or ketones products of oxidative deamination are on the right. 5-HIA, 5-hydroxyindoleacetaldehyde

DDC, also known as aromatic amino acid decarboxylase (AADC), has a broad substrate specificity being able to convert both L-dopa and L-5-hydroxytryptophan to dopamine and serotonin (Fig. 3a), respectively, in addition to other aromatic amino acids, producing the so-called trace amines (Bertoldi 2014). A scheme of all reactive substrates or products of DDC is reported in Fig. 4. A decarboxylation-dependent transamination was identified as a side reaction for DDC (O’Leary and Baughn 1977). This reaction consists of an off-pathway step following decarboxylation at Cα (Fig. 1) where protonation of Q1 occurs at C4′ instead of at Cα leading to equimolar amount of carbonyl compound and pyridoxamine 5′-phosphate (PMP), a coenzyme incapable of performing further decarboxylation and thus leading to enzyme inactivation. However, experimental data in the presence of α-methyldopa did not support this reaction mechanism, since the produced carbonyl compound far exceeded, on a molar basis, coenzyme content (Barboni et al. 1981). This behavior remained obscure until the end of last century, when our group has demonstrated that mammalian DDC catalyzes an oxidative deamination reaction with serotonin, dopamine, and α-methylDopa, following α-methyldopamine formation, concomitantly with oxygen consumption (Bertoldi et al. 1996, 1998, 1999b; Bertoldi and Borri Voltattorni 2003). These reactions are accompanied by enzyme inactivation through different pathways, mechanism-based (Bertoldi et al. 1996) or affinity labeling (Bertoldi et al. 1998) due to the covalent binding of the carbonyl product to the PLP coenzyme. This inactivation was interpreted as a possible regulation of DDC concurring in exacerbating oxidative stress conditions (see below). The proposed mechanism for oxidative deamination starts with PLP forming an external aldimine with the aromatic amine that is subsequently deprotonated, giving a quinonoid species accessible to oxygen, then forming the carbonyl compound, ammonia, and consuming oxygen. A small amount of PMP was produced, so the occurrence of the decarboxylation-dependent transamination (also called abortive transamination in some papers) was not excluded (Fig. 3a). It was also assessed that, in the absence of oxygen, aromatic amines undergo a half-transamination reaction (Bertoldi and Borri Voltattorni 2003), whereas α-methyldopa underwent the decarboxylation-dependent transamination (Bertoldi and Borri Voltattorni 2000), leading, in both cases, to the conversion of PLP into catalytically inactive PMP and a stoichiometric amount of the aldehyde/ketone. Thus, DDC has the ability to protonate the quinonoid intermediate at C4′ and not only at Cα (Fig. 3a).

It has also been demonstrated that the main decarboxylation reaction of DDC is somewhat sensitive to oxygen, since in its absence, both *k*_cat_ and *K*_m_ for l-Dopa and L-5HTP are decreased and PMP is formed, due to a decarboxylation-dependent transamination (Bertoldi and Borri Voltattorni 2000), reinforcing a possible regulation mechanism exerted by O_2_. Until these findings, DDC was supposed to catalyze only paracatalytic reactions with oxygen.

In this complex picture, it was unexpected that mutation of Tyr-332, a conserved residue, to phenylalanine transforms DDC into a decarboxylation-dependent oxidative deaminase, that is an enzyme not able to catalyze amine formation, rather Y332F reacts with oxygen and forms 3,4-dihydroxyphenylacetaldehyde (DHPAA or DOPAL) and ammonia (Fig. 3b) pointing out a catalytic role for Tyr-332 in the main decarboxylation mechanism as the residue responsible for the Cα protonation (Bertoldi and Voltattorni 2001; Bertoldi et al. 2002). This residue belongs to a mobile loop of DDC presumed to play important roles in the activity of the enzyme, since it is predicted to cover the active site as a lid positioning essential residues in a proper orientation and microenvironment useful for catalysis (Paiardini et al. 2017). Under anaerobic conditions, the reaction of the Y332F DDC with its substrates immediately converts PLP into PMP (Bertoldi et al. 2002). These observations pushed the investigation towards the identification of intermediates of the oxygen-consuming reaction catalyzed by DDC. A substrate analog, d-tryptophan methyl-ester, that mimics the position of the α-proton of the aromatic amines with its stereospecificity, allowed the identification and validation of a quinonoid species as an intermediate of the oxidative deamination reaction (Bertoldi et al. 2005), thus confirming what was hypothesized by Abell and Schloss about the reactivity of the carbanionic intermediate with oxygen. The step forward has been to elucidate what happens in the steps following oxygen binding to the quinonoid. An insight came from further investigations leading to the identification and characterization of other intermediates of the oxidase activity, so that a chemical mechanism could be advanced (Bertoldi et al. 2008), where it emerged how oxygen is able to control the equilibrium among reaction intermediates of DDC.

**Fig. 5:**
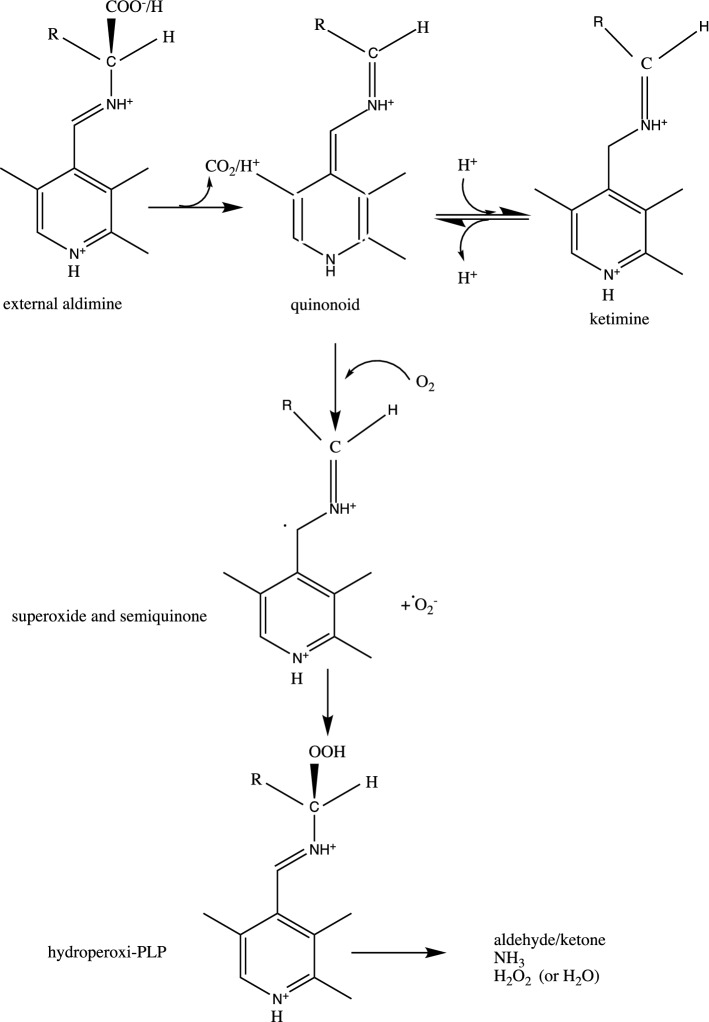
Reaction mechanism of oxidative deamination catalyzed by DDC. The substrates of the reaction with oxygen (both aromatic amino acids or amines) at first form an external aldimine that is subsequently decarboxylated (from amino acids) or deprotonated (from amines) to give the quinonoid that partitions between C4′ protonation to give a ketimine that is in equilibrium with the quinonoid that could be attacked by oxygen generating a radical pair, semiquinone, and superoxide anion, that form a hydroperoxy-PLP intermediate responsible for the synthesis of the oxidative deamination products. R refers to the aromatic (cathecol or indole) moiety. (Adapted from (Bertoldi et al. 2008))

Overall, a reaction mechanism of DDC with O_2_ has been finally accepted (Fig. 5) (Bertoldi et al. 2008). Once the external aldimine is formed, decarboxylation or deprotonation takes place leading to a quinonoid species in equilibrium with a ketimine species that forms upon reprotonation to C4′. The presence of oxygen controls this equilibrium in favor of the quinonoid species that is attacked by O_2_ giving rise to a radical one-electron transfer and forming superoxide anion and semiquinone. Superoxide couples with semiquinone generating a hydroperoxide species. From this intermediate, the products of the reaction are then generated.

## Other aromatic amino acid decarboxylases reactive towards oxygen: AADCs and aromatic aldehyde synthases (AAS)s

AADCs from other different sources (plants, mushrooms, and insects) are known, since these enzymes have evolved in many organisms yielding a number of paralogous enzymes with different substrate specificity and catalytic mechanisms (Facchini et al. 2000; Torrens-Spence et al. 2020).

**Fig. 6:**
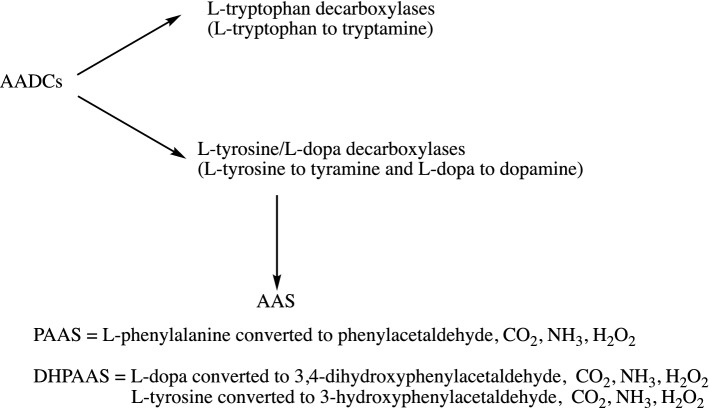
Plant AADCs and AASs. Plant AADCs are specialized enzymes clustered in two groups: l-tryptophan decarboxylases producing tryptamine and l-tyrosine/l-dopa decarboxylase producing tyramine/dopamine. A different group probably originating from l-tyrosine/l-dopa decarboxylases identifies AASs such as PAAS and DHPAAS converting aromatic amino acids into the corresponding aromatic aldehydes, ammonia and hydrogen peroxide, and consuming oxygen

The plant kingdom is particularly specialized, since plant AADCs, differently from those of mammals and insects, are characterized by a narrow substrate specificity, exhibiting a substrate preference for either phenol or indole amino acid, giving rise to l-tyrosine/l-dopa decarboxylases or to l-tryptophan decarboxylases (Noé et al. 1984; Facchini and De Luca 1994; Facchini et al. 2000). These enzymes, isolated from a variety of plant species (Facchini et al. 2000), produce aromatic monoamines acting as precursors for many important natural products useful to plant metabolism. No activity towards oxygen has been reported for these enzymes (Fig. 6).

In 2006, a paper reported the identification of a tomato PLP-l-phenylalanine decarboxylase able to remove CO_2_ and produce phenethylamine, subsequently converted into phenylacetaldehyde by an identified enzymatic activity in the pathway of 2-phenylethanol, a compound essential to contribute to tomato flavor (Tieman et al. 2006). In the same year, the identification of rose and petunia phenylacetaldehyde synthase (PAAS) has been reported. These PLP-dependent enzymes show high amino acid identity (about 65%) with plant l-tyrosine/l-dopa decarboxylases and l-tryptophan decarboxylases, and directly convert l-phenylalanine into phenylacetaldehyde by a decarboxylation followed by an oxidative deamination, consuming oxygen without accumulation of the amine (Kaminaga et al. 2006). Other aromatic aldehyde synthases (AAS) have been identified and characterized in the following years, even if it should be mentioned that, originally, these enzymes have been annotated as aromatic amino acid decarboxylases and not accurately investigated (Fig. 6). Then, it was assessed that PAAS enzymes represent a subgroup of l-tyrosine/l-dopa decarboxylases (Torrens-Spence et al. 2012), exhibiting high activity not only with l-phenylalanine but also with l-dopa and l-tyrosine: *Arabidopsis* PAAS seems to play a role in wounding/herbivory response and flower scent production (Gutensohn et al. 2011), while parsley PAAS synthesizes 4-hydroxyphenylacetaldehyde to enhance its stress/defense response (Torrens-Spence et al. 2012) (Fig. 6).

In an elegant paper, Torrens-Spence and coauthors have demonstrated by site-directed mutagenesis that a true AADC could be converted into AAS and vice versa by changing the chemical identity of a conserved residue (Torrens-Spence et al. 2013). A role as a signature residue has been proposed for a tyrosine of the mobile loop near the active site (which aligns with tyrosine-332 of mammalian DDC) that can be substituted by a phenylalanine in AADC in different plants. Interestingly, the presence of tyrosine gives rise to decarboxylases that produce aromatic amines, while that of phenylalanine gives decarboxylation-dependent oxidative deaminating enzymes (Torrens-Spence et al. 2013), reminiscent of the switch in activity displayed by the Y332F mammalian DDC (Bertoldi et al. 2002). The tyrosine residue has been suggested not to directly protonate the α-carbon to generate the amine product as it does for the mammalian DDC (Bertoldi et al. 2002); rather, it has been proposed that an active site histidine forms an hydrogen bond with the loop tyrosine that plays an indirect role in stabilizing the histidine side chain to the protonation of the α-carbon (Torrens-Spence et al. 2013). This is not the case for mammalian DDC, since mutation of the same histidine (His-192) to asparagine leads to a variant able to perform both decarboxylation with production of amine and decarboxylation-dependent oxidative deamination of aromatic amines (Bertoldi et al. 2001).

Insects use dopamine, in addition to its role as a neurotransmitter, in metabolic processes leading to cuticle sclerotization. In this context, AADC enzymes (Fig. 6) are known to play an important role. However, AADC from *Aedes aegypti*, with 70% sequence homology with the mammalian enzyme, has been demonstrated to catalyze the direct oxidation of l-dopa to 3,4-dihydroxyphenylacetaldehyde (DHPAA or DOPAL) bypassing dopamine formation, and is thus classified as DHPAA synthase (DHPAAS) (Vavricka et al. 2011). The role of aromatic aldehyde formation could be related to cross-linking complexes involved in cuticle hardening.

The mechanism of *Drosophila* DHPAAS has been extensively investigated and a catalytic role to the active site residue Asn-192 has been proposed, since its change to histidine leads to the partial conversion of DHPAAS to a classical decarboxylase (amine formation) rather to aldehyde synthase. On this basis, His-192 in AADCs of insects has been appointed as the residue responsible for quinonoid α-carbon protonation. However, since the mutational analysis did not give rise to enzymatic species completely devoid of one of the two activities, the real effect of this residue (histidine or asparagine) appears difficult to be precisely defined (Liang et al. 2017).

In fungi, a few papers refer to AADC activity (Fig. 6), mainly related to the decarboxylation of l-tryptophan (Kalb et al. 2016). An in-depth investigation has been carried out in *Psilocybe cubensis*, a mushroom responsible for psychoactive alkaloid psilocybin synthesis, revealing the presence of both a DHPAAS with a phenylalanine instead of a tyrosine in the catalytic loop and an AADC-like protein (similar to plant l-tryptophan decarboxylases) (Torrens-Spence et al. 2018). Interestingly, in this AADC enzyme, a regulation by calcium ion was reported. (Torrens-Spence et al. 2018).

## The reactivity of other fold-type I PLP decarboxylases with oxygen

In the same years, it was demonstrated that among PLP enzymes, the PLP-dependent decarboxylases are particularly able to perform oxidative reactions with O_2_, in addition to their main reaction, maybe for their intrinsic possibility of exchanging CO_2_ with O_2_. It is not unprecedented that, just like RuBisCO, an enzyme able to exchange a gas like CO_2_ could be prone to accept O_2_, even this does not justify its reactivity.

By the way, fold-type I (or a-aspartate aminotransferase family) (Grishin et al. 1995) amino acid decarboxylases [that from an evolutionary point of view are clustered in Group II α-decarboxylases (Sandmeier et al. 1994)] are responsible for the synthesis of essential biomolecules, such as polyamines and hypotaurine, or neurotransmitters, such as dopamine, serotonin, and γ-aminobutyric acid (GABA) (Paiardini et al. 2017). They comprise aromatic amino acid, glutamate, and histidine decarboxylases from all sources together with the prokaryotic ornithine and lysine decarboxylases.

**Fig. 7:**
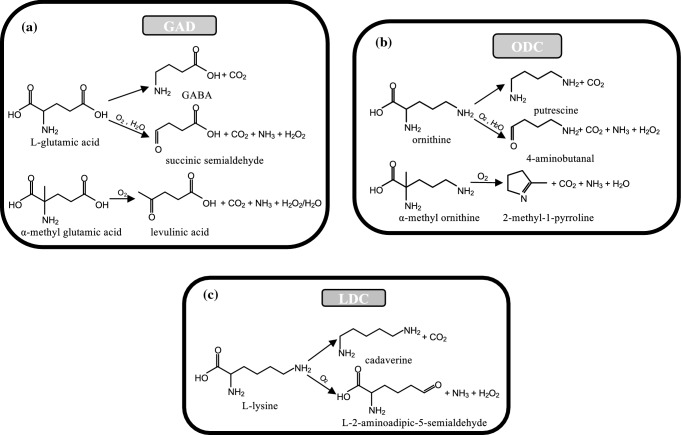
Reactions of PLP α-decarboxylases. **a** GAD with l-glutamic acid and a-methylglutamate, **b** ODC with l-ornithine and α-methylornithine, and** c** LDC with l-lysine

The main activities played by these enzymes together with the new oxidase reactions are reported in Fig. 7. *E. coli* glutamate decarboxylase (GAD), in addition to the decarboxylation of l-glutamic acid into GABA, transforms glutamate to succinic semialdehyde (Abell and Schloss 1991) and α-methylglutamate to levulinic acid (Bertoldi et al. 1999a) (Fig. 7a). There are some signs that corroborate the possibility for both human GAD65 and GAD67 to interact with oxygen; in fact, GAD65 produces succinic semialdehyde from glutamate, even if it was not demonstrated that oxygen is required (Choi and Churchich 1986; Bunik et al. 2011).

Ornithine decarboxylase (ODC) from different prokaryotic sources, such as *Lactobacillus 30a* or *Hafnia alvei*, has been demonstrated to catalyze, in addition to putrescine formation, a decarboxylation-dependent oxidative deamination (Bertoldi et al. 1999a; Sakai et al. 1997) of both ornithine and α-methylornithine, in a reaction directly converting the amino acid into the corresponding carbonyl compound and ammonia, without accumulation of the amine (Fig. 7b).

An l-lysine decarboxylase (LDC) from *Burkholderia* sp. AIU 395 has also been found to catalyze both α-decarboxylation, giving cadaverine, and an oxidative ε-deamination, giving L-2-aminoadipic 5-semialdehyde (Sugawara et al. 2014) (Fig. 7c).

## Biological roles of oxidative side reactions of human PLP-dependent decarboxylases

**Fig. 8:**
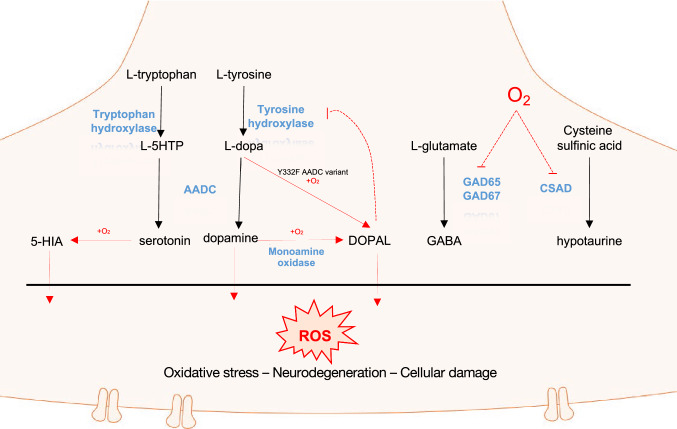
Oxidative reactions played by mammalian PLP decarboxylases and their possible interplay. The metabolic pathways of aromatic amino acids and amine metabolism start with hydroxylation performed by tryptophan and tyrosine hydroxylase on the corresponding amino acids, and then, AADC decarboxylates L-5HTP and L-dopa to the corresponding amines serotonin and dopamine that in the presence of oxygen are oxidatively deaminated to the corresponding aromatic aldehydes, 5-HIA and DOPAL. Oxygen also influences mammalian GAD and CSAD activity by inhibiting these enzymes. Altogether, oxidative products (carbonyl compounds and ROS) could concur in exacerbating oxidative stress conditions and contributing to worsen neurodegeneration and cell damages. Monoamine oxidase is the known enzyme deputed to oxidase amine to aldehyde in an FAD-dependent manner. In black, amino acids amines and metabolic pathways, in red oxygen-related reactions, and in blue the name of the enzymes

The interpretation given for the O_2_ interaction of PLP-dependent decarboxylases can be at least partially linked to pathological states or to a physiological regulation of the activities of some important brain enzymes (Fig. 8). It may be discussed if this catalytic activity could represent a mechanism of regulation of neurotransmitter synthesis or catabolism, and this fact could be of particular interest for diseases characterized by a dysregulation in neurotransmitters, such as Parkinson’s Disease, AADC deficiency, pyridoxine oxidase-related epilepsy, and oxygen-induced seizures. The interplay of PLP decarboxylases with dioxygen may be involved in and regulate oxidative/metabolic stress in human brain diseases (Bunik et al. 2007). In addition, a survey regarding oxidative paracatalytic reactions catalyzed by enzymes able to form carbanionic intermediates, including also some PLP-dependent enzymes, appeared in 2011 (Bunik et al. 2011). In this review, the authors pointed out the effects exerted by these side reactions involving oxygen especially in neurodegeneration and cancer. Regarding PLP enzymes, Bunik et al. highlighted that human AADC’s ability to produce DHPAA/DOPAL, even possibly increased in altered forms of the enzyme, can result in pathology due to the known neurotoxicity of this compound, linked to Parkinson’s disease neurodegeneration of the *substantia nigra* neurons (Burke et al. 2003). DOPAL neurotoxicity mechanisms, indeed, included DOPAL-α-synuclein cross-linked oligomerization (Follmer et al. 2015) as well as extensive DOPAL-induced protein-quinone adduct formation (“quinonization”) and protein oligomerization, ubiquitination, and aggregation (Jinsmaa et al. 2018). Also 5-hydroxyindolacetaldehyde (5-HIA), produced by mammal AADC from serotonin ((Bertoldi et al. 1996), was demonstrated to promote α-synuclein oligomerization both in vitro and in a synuclein-overexpressing cell model, in a manner similar to DOPAL (Jinsmaa et al. 2015), possibly explaining the early degeneration of dorsal raphe nuclei serotonergic neurons in Parkinson’s disease. Interestingly, among DOPAL-modified/inhibited targets, tyrosine hydroxylase (TH) shows a semi-reversible mechanism with consequent decrease in overall L-Dopa production, but a time- and concentration-dependent TH activity recovering was observed upon removal of DOPAL (Mexas et al. 2011). This DOPAL-dependent reversible inhibition of TH could provide a new perspective in AADC ability to perform oxidative deamination, linking this side reaction to a possible physiological feed-back mechanism by which a minor AADC product can regulate l-dopa production targeting the rate-limiting enzyme in the dopamine synthetic pathway.

Both human recombinant GAD65 and GAD67 were shown to be potently and reversibly inhibited by molecular oxygen. In particular, GAD65-O_2_ dependent inhibition has been linked to the genesis of oxygen-induced seizures (Davis et al. 2001) by limiting GABA production and loading in vesicles (Hsu et al. 1999) and in the pathogenesis of type I diabetes mellitus (Trigwell et al. 2001). On the other side, the isoform GAD67 was shown to be related with convulsions induced by hyperbaric oxygen exposure both in vivo and in vitro (Li et al. 2008).

Like GAD65, also cysteine-sulfinic acid decarboxylase (CSAD) has been reported to be responsive to reversible inhibition by O_2_ (Davis et al. 2001) (Fig. 8). Cysteine sulfinic acid decarboxylation product hypotaurine is then oxidized to taurine by an oxygenase, with the latter compound shown to be especially important in central nervous system, where it plays a wide range of different roles (Menzie et al. 2014) and is linked to early retinal degeneration (Preising et al. 2019). As such, inhibition of CSAD by O_2_ could contribute to retinal damage pointed out in premature infants exposed to high O_2_ levels at birth (Jewell et al. 2001). Noteworthy, these two decarboxylases are also inhibited by nitric oxide (Davis et al. 2001), another radical-containing compound as dioxygen, suggesting a possible radical mechanism in the inhibition. Reactions with species such as O_2_ or NO could, in fact, proceed through a one-electron stepwise transfer eventually leading to radical intermediates, as mentioned before.

The interaction of PLP-dependent decarboxylases with molecular oxygen opens the way to the possibility that these enzymes could be strongly involved in oxidative damage, since the oxidase activity is linked to the concomitant production of hydrogen peroxide (Bunik et al. 2007). In this scenario, an enzyme producing ROS and carbonyl compounds could exacerbate oxidative stress conditions (Bunik et al. 2007). Moreover, it cannot be excluded that the proven sensitivity to or interaction with oxygen of these PLP-dependent decarboxylases could be extended to ROS compounds, underlying the possibility that these enzymes can be inactivated and/or form ROS-driven adducts with other proteins, and the consequent necessity of monitoring them as targets of ROS themselves. A broader effect of the oxidative reactions of PLP-dependent decarboxylases, responsible for neurotransmitters synthesis, could be the interplay between ROS and the important modulator nitric oxide which, in turn, leads to the production of nitrogen reactive species contributing to nitro-oxidative stress. Its synthesis and regulation involves a network of pathways that should be taken into consideration (Persichini et al. 2016).

The emerging question is how the propensity to react with oxygen is linked to inhibition exerted by the products of the oxidative paracatalytic reactions and their regulation in PLP decarboxylases. Although it is not easy to address such issue, an important review (Schloss 2002) interprets the oxygenase reaction of GAD as a consequence of a possible radical character also of the main decarboxylation reaction of the enzyme gaining assistance also in the main activity played by the enzyme.

## New PLP enzymes of fold-type I are able to catalyze reactions involving O_2_

In the last 5 years, a growth in papers reporting new oxygen-consuming PLP enzymes has occurred. Most of them belong to the bacterial or fungal kingdoms and can be grouped by the fact that dioxygen is a substrate of their main reaction.

An excellent recent review gives extensive details about the oxidative reaction catalyzed by each one of these new enzymes (Hoffarth et al. 2020). In the present review, we will briefly review the activity catalyzed and the functional role.

Three of these new PLP enzymes have the common feature of acting on l-arginine as substrate and could catalyze the four-electron oxidation of l-arginine, with a mechanism involving either an oxidation or a hydroxylation, that are similar oxidative pathways undergoing through different fates of the carbonyl products.

**Fig. 9:**
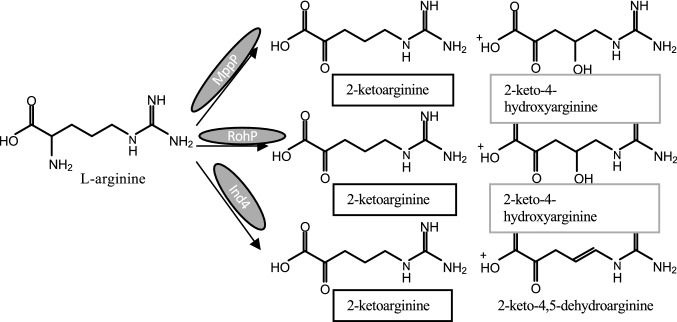
Main reactions catalyzed by other PLP-dependent enzymes, MppP, RohP, and Ind4, in the presence of l-arginine

*Streptomyces wadayamensis* MppP is a PLP enzyme that catalyzes the hydroxylation and deamination of l-arginine consuming molecular oxygen and producing the carbonyl compounds 2-ketoarginine and 2-keto-3,4-dehydroarginine in a 1:1.7 ratio (Fig. 9) (Han et al. 2015). The latter is an essential guanidino acid for the synthesis of bioactive peptides such as l-enduracididine (Han et al. 2015), which is a cyclic analog of arginine found in several antimicrobial peptides useful for treatment of drug-resistant pathogens. The structure of MppP reveals that it is a homodimer, belonging to the fold-type I of PLP enzymes, as the a-decarboxylases reported above, with the active sites present at the interface between the two subunits; however, unlike other aminotransferase family enzymes, in MppP, the contribution of the opposite chain to each active site is minimal (Han et al. 2015). An in-depth analysis of the catalytic activity of this enzyme was published subsequently (Han et al. 2018) and reported the exact stoichiometry of all products formed. A refined chemical mechanism of this PLP-driven oxidase activity together with a structural analysis was proposed, highlighting the role of the N-terminus switching from an ordered to a disordered state during binding/catalysis.

Another PLP enzyme, RohP, contributes to the biosynthesis pathway of azomycin (Hedges and Ryan 2019) and acts on l-arginine catalyzing an oxygen-consuming reaction leading to both hydroxylation and deamination (Fig. 9) (Hedges et al. 2018). RohP is from *Streptomyces cattleya* and presents a high sequence identity with both MppP and Ind4 (see below) (Hedges et al. 2018). From a functional point of view, RohP acts similarly to MppP, forming an identical product, i.e., a hydroxylated ketoacid.

Finally, *Streptomyces griseus* Ind4, a PLP enzyme of the indolmycin biosynthetic pathway catalyzing a reaction similar to those of MppP and RohP, thus converts l-arginine into an oxidized product (Du et al. 2016). The peculiarity of this reaction is that Ind4 forms a C = C bond rather than a carbonyl hydroxylated compound as do MppP and RohP (Fig. 8) even if 2-ketoarginine is produced.

**Fig. 10:**
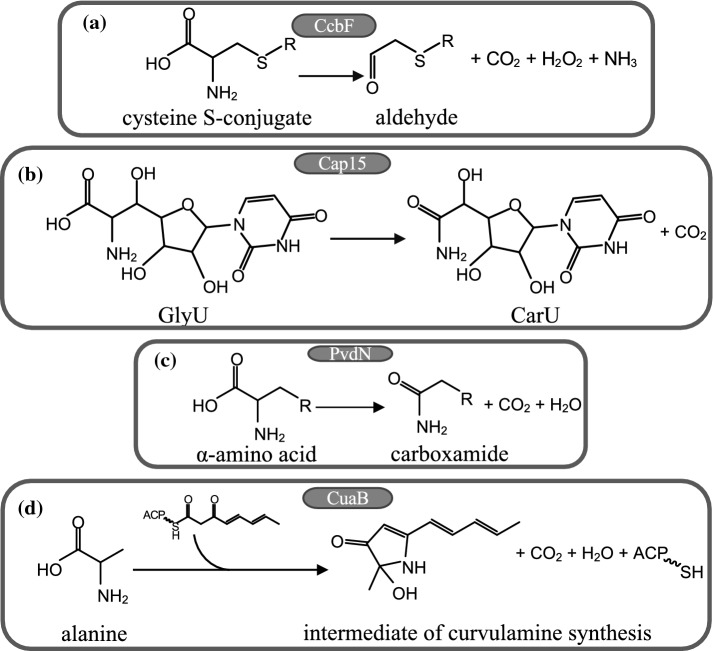
Main reactions catalyzed by other PLP-dependent enzymes on bioactive compounds. **a** CcdF on a cysteine-S-conjugate, **b** Cap15 on GlyU, **c** PvdN, and **d** CuaB on more complex amino acidic substrates

Another PLP enzyme capable of consuming molecular oxygen is *E. coli* CcbF. It belongs to the metabolic pathway of lincosamide antibiotics, compounds that act on the large subunit of bacterial ribosomes blocking protein synthesis (Wilson 2014). In this process, CcbF catalyzes the transformation of a cysteine S-conjugate into an aldehyde with co-production of carbon dioxide, hydrogen peroxide, and ammonia in the presence of molecular oxygen which is concomitantly consumed (Fig. 10) (Wang et al. 2016). In this sense, the enzyme is more similar to a decarboxylation-dependent oxidative deaminase rather than to an enzyme that oxidizes an inactivated carbon–carbon bond (Wang et al. 2016).

The following three enzymes have been classified as catalyzing a decarboxylating oxygenation (Hoffarth et al. 2020); however, some differences should be pointed out. PvdN is a PLP enzyme of *Pseudomonas* involved in pyoverdine biosynthesis. From a structural point of view, it belongs to the fold-type I of PLP enzymes (Drake and Gulick 2016). A recent investigation on its functional role suggests that it catalyzes the decarboxylation of its substrate generating a quinonoid intermediate that subsequently undergoes an oxygenase reaction leading to the products through a hydroperoxide species (Fig. 10) (Ringel, Dräger and Brüser 2016). The spatial structure shows two tunnels from each active site to the protein surface: a larger one accommodating the substrate and a narrower one for the possible exchange of CO_2_ and O_2_ (Drake and Gulick 2016). In no other structure of decarboxylases, catalyzing a decarboxylation and a successive oxygenation has such a channel been identified.

Cap15 is a *Streptomyces* PLP-dependent a-amino acid monooxygenase–decarboxylase acting in the biosynthetic pathway of capuramycins, a class of antimycobacterial antibiotics (Huang et al. 2018). The reaction catalyzed is the conversion of the unusual b-hydroxy-a-amino acid 5′-C-glycyluridine (GlyU) into the related carboxamide product CarU (Fig. 10). The novelty of such activity is that the catalytic mechanism proceeds by at first the deprotonation of the external aldimine intermediate, followed by oxygenation of the quinonoid species, generating a radical pair of hydroperoxy-PLP superoxide anion finally generating water and carbon dioxide (Huang et al. 2018), thus exclusively for this enzyme, decarboxylation occurs after oxygenation.

Recently, a new PLP enzyme, CuaB, has been identified in the gene cluster for the synthesis of an indolizidine alkaloid, curvulamine, in fungi (Dai et al. 2020), underlining the fact that the reactivity of PLP enzyme with oxygen is a widespread feature. CuaB is a bifunctional enzyme: it catalyzes the formation of C–C bonds through Claisen condensation, as other PLP enzymes are able to do, such as 8-amino-7-oxononanoate synthase and, unexpectedly, a hydroxylation using dioxygen (Fig. 10). A detailed enzyme mechanism has been proposed on the basis of the collected products (Dai et al. 2020). The external aldimine with the substrate is deprotonated to generate a quinonoid that follows the pathway of synthases (Fig. 1). Once the external aldimine with the product is formed, it is decarboxylated generating a second quinonoid reactive towards oxygen forming a hydroperoxide that is converted into a hydroxylated product and water. Interestingly, this sequential reaction with the product is reminiscent of the activities of DDC with serotonin, dopamine, and α-methyldopamine (Bertoldi et al. 1996, 1998, Bertoldi and Borri Voltattorni 2003).

The identification of these enzymes mainly in *Pseudomonas* and *Streptomyces* is perhaps related to investigations on bioactive compounds and it can be hypothesized that many other PLP enzymes are dispersed in the living kingdoms whose activities are still unrecognized. The ability to couple PLP and oxygen chemistry not only for paracatalytic reactions but also in main enzymatic reactions broadens PLP-enzyme functional ability and paves the way for further studies.

## A structural basis for oxygen reactivity of decarboxylases

This review points out that the reactivity with dioxygen, at first considered as a mere artifact of the carbanionic chemistry of some PLP decarboxylases, is more common than expected. From a structural point of view, it seems to be related not only to decarboxylases, but also to other fold-type I enzymes: however, it should be noticed that the majority of PLP enzymes consuming oxygen catalyze a decarboxylation reaction. While an oxidase activity could be related to a regulation role or alteration of the physiological state in mammalian or plant decarboxylases, it is not easy to envisage a role in other organisms such as bacteria or fungi. Literature on this topic is still lacking, maybe due to recent identification of such oxygen-consuming activities in PLP enzymes of these organisms.

The fact that this oxidase activity remains confined to enzymes of the aminotransferase family could be due, on one hand, to the fact that this family is the most abundant among PLP enzymes, on the other, to the fact that fold-type I enzymes could possess some structural element or active site conformation more prone to oxygen entrance.

There is as yet no evidence for oxygen-dependent reactions in the fold-type III decarboxylases, a large group containing eukaryotic lysine, eukaryotic ornithine, prokaryotic and eukaryotic arginine, and prokaryotic diaminopimelate decarboxylases (Grishin et al. 1995). Oxygen is not reported to react with PLP enzymes even belonging to other fold-types.

It could be interesting to compare the structural differences of the solved spatial arrangements of prokaryotic (Momany et al. 1995) and eukaryotic ODC (Kern et al. 1999) as prototypes of fold-type I and III decarboxylases, respectively. One of the main differences is the orientation of the reactive group on the external aldimine with respect to the buried or exposed face of the cofactor. In fold-type I enzymes, the lysine residue forming the internal aldimine is on the *si* face buried in the interior of the protein, while the opposite *re* face is exposed. When this intermediate is converted into the external aldimine, the carboxylate moiety protrudes on the buried *si* face and reaction then proceeds. Instead, in fold-type III enzymes, the coenzyme has a mirrored position and the lysine of the internal aldimine is buried on the *re* face of the cofactor, while the *si* face is exposed. The external aldimine thus presents the CO_2_ on the exposed *si* face (Kern et al. 1999). This fact has been linked to stereospecificity of catalysis (Kern et al. 1999); however, it can be argued that dioxygen could bind and react better in a buried active site. A second structural feature is the dimer stability much higher in fold-type I ODC than in fold-type III ODC (Kern et al. 1999).

An elegant example of enzymes catalyzing the same reaction but belonging to different fold-types is also found in transaminases. The L-amino acid transaminases belong to fold-type I, while d-amino acid transaminase belong to fold-type IV (Grishin et al. 1995) and the differences are the PLP location in the active site and the dimer assembly (Humble et al. 2012).

From a functional point of view, there is evidence that diaminopimelate decarboxylase and d-ornithine/d-lysine decarboxylase have a concerted decarboxylation/protonation step, ruling out the quinonoid intermediate formation (Fogle and Toney 2011; Phillips et al. 2019). This could explain the lack of O_2_ reactivity. However, the structural determinants responsible of this concerted step are presently still unknown. Whatever the structural difference could be, the identification in PvdN of the putative O_2_ tunnel could foster re-evaluation of already deposited structures of decarboxylases for the presence of any potential channels.

## Conclusions

The oxygen reactivity of PLP enzymes could be of great physiological interest, given the fact that it seems typical of enzymes producing important neurotransmitters or neuromodulators, whose effect could be greatly affected by redox conditions. Following this view, it would be interesting to further investigate the influence of oxygen on activity and inhibition of these enzymes.

The new biosynthetic pathways described in the last few years, mainly in bacteria and fungi, increase the evidence of the high catalytic versatility of PLP enzymes in nature. These activities have been generally identified by the following genome sequence-based survey. The approach of characterizing the potentially diverse activities arising from the ductile nature of PLP chemistry would allow access to unpredictable new biological processes.

## Abbreviations

PLP: Pyridoxal 5′-phosphate
AADC or DDC: Aromatic amino acid or dopa decarboxylase
GAD: Glutamate decarboxylase
DHPAA or DOPAL: 3,4-Dihydroxyphenylacetaldehyde
DHPAAS: 3,4-Dihydroxyphenylacetaldehyde synthase
PAAS: Phenylacetaldehyde synthase
GABA: γ-Aminobutyric acid
ODC: Ornithine decarboxylase
LDC: l-Lysine decarboxylase
ROS: Reactive oxygen species
TH: Tyrosine hydroxylase
